# Tumor suppressive role of miR-33a-5p in pancreatic ductal adenocarcinoma cells by directly targeting RAP2A

**DOI:** 10.1186/s11658-021-00265-w

**Published:** 2021-06-05

**Authors:** Yanfen Lian, Dongxiao Jiang, Jiangtao Sun

**Affiliations:** Department of Oncology, Weihai Central Hospital Affiliated to Medical College of Qingdao University, No.3, West Mishan East Road, Weihai, Shandong China

**Keywords:** PDAC, miR-33a-5p, RAP2A, Proliferation, Migration, Invasion

## Abstract

**Background:**

The suppressive effects of miR-33a-5p have been reported in colorectal cancer and lung cancer. However, the functional role of miR-33a-5p in pancreatic ductal adenocarcinoma (PDAC) has not yet been elucidated.

**Methods:**

The expression of miR-33a-5p was determined using reverse-transcription quantitative PCR (RT-qPCR) in PDAC tissues and cell lines. The association between miR-33a-5p expression and clinical categorical parameters was analyzed by the chi-square test. Cell proliferation was analyzing by Cell Counting Kit -8 (CCK-8) assay. Transwell assay was utilized to assess cell migration and invasion. The interactions between miR-33a-5p and RAP2A were verified by luciferase reporter assay, RT-qPCR, western blot analysis and RNA immunoprecipitation (RIP) assay.

**Results:**

Here, we observed for the first time that miR-33a-5p expression level was significantly decreased in PDAC tissues and cell lines. There was a significant association between decreased miR-33a-5p expression and TNM stage or lymph node metastasis. Overexpression of miR-33a-5p significantly inhibited SW1990 and PANC-1 cell proliferation, migration and invasion. Knockdown of miR-33a-5p remarkedly promoted cell proliferation, migration and invasion in BxPC-3 and ASPC-1. Mechanistically, RAP2A was confirmed as the target of miR-33a-5p in PDAC cells. Moreover, RAP2A overexpression abolished miR-33a-5p-mediated suppressive effects on SW1990 and PANC-1 cells.

**Conclusions:**

Taken together, these results suggest that miR-33a-5p exerted tumor suppressive effects on PDAC cells by targeting RAP2A, which might provide a new theoretical basis for the clinical treatment of PDAC.

## Background

Pancreatic ductal adenocarcinoma (PDAC) is one of the leading causes of tumor-associated deaths worldwide [1, 2]. Several risk factors, such as advanced age, smoking, obesity, and chronic pancreatitis, are reported to be associated with PDAC development [3–5]. Despite great achievements have been made in current treatment regimens, including surgical resection, chemotherapy and radiation therapy, the survival prognosis of PDAC patients still remains poor, with a five-year survival rate lower than 7% [6]. Thus, exploration of the molecular mechanisms underlying PDAC cellular functions is urgently needed to develop effective therapeutic strategies.

As a class of small endogenous non-coding RNAs with 19–22 nucleotides, microRNAs (miRNAs/miRs) are major regulators of diverse cellular functions, including cell differentiation, proliferation and metastasis [7, 8]. Mechanically, miRNAs usually cause mRNA degradation and/or translational inhibition by targeting mRNAs via directly binding to their 3′-untranslated regions (3′-UTRs) [9]. MiRNAs have been widely reported to be aberrantly expressed and play important roles in the development of cancer, including PDAC [10–12]. Recent studies showed that miR-33a (consisting of two single stranded miRNAs: miR-33a-5p and miR-33a-3p) is located in intron 16 of chromosome 22, which is involved in cholesterol biosynthesis and cholesterol uptake [13]. Importantly, miR-33a acts a tumor suppressor via regulating cellular functions in several human cancers. For instance, overexpression of miR-33a-5p significantly inhibited cell growth of osteosarcoma [14]. Up-regulation of miR-33a-5p impaired cell proliferation and metastasis of esophageal squamous cell carcinoma (ESCC) cells [15]. In addition, the suppressive effects of miR-33a-5p on cell proliferation and migration on colorectal cancer [16], drug-resistant triple-negative breast cancer (TNBC) [17] and lung cancer [18] have been demonstrated. Nevertheless, the functional role of miR-33a-5p has not yet been reported in PDAC.

Rap proteins (RAP1A, RAP1B, RAP2A, RAP2B and RAP2C) belong to the small GTPase superfamily, members of which are involved in a variety of biological processes [19, 20]. RAP2A, as a member of the Ras superfamily, was reported to be upregulated in lung cancer [21] and nasopharyngeal carcinoma [22]. Moreover, Wu et al. [23] showed the accelerative effects of RAP2A overexpression on renal cell carcinoma cell migration and invasion. Wu et al. [24] further confirmed that ectopic expression of RAP2A promoted cancer cell metastasis in vitro by elevating p-Akt expression. Based on our previous prediction that miR-33a-5p exhibits targeted regulation of RAP2A expression, we thus speculated that the miR-33a-5p/RAP2A axis might be an important regulator in the development of PDAC.

To validate our hypothesis, this study was designed to determine the miR-33a-5p expression levels in PDAC tissues and explore their association with clinical categorical parameters. Then, we investigated the effects of miR-33a-5p on cell proliferation, migration and invasion. We further confirmed whether miR-33a-5p exerted these effects via suppression of RAP2A in PDAC cells. Our findings might provide a new theoretical basis for the clinical treatment of PDAC.

## Materials and methods

### Patients and clinical samples

A total of 58 pairs of PDAC tissues and matched adjacent tissues were obtained at Weihai Central Hospital Affiliated to the Medical College of Qingdao University (Shandong, China). Prior to surgery, none of the patients had received radiation or chemotherapy and all had signed written informed consent. The clinical characteristics of these patients are listed in Table 1. All collected tissues were kept at − 80 °C for further use. This study was conducted in accordance with the Declaration of Helsinki (1975), which obtained approval from the Ethics Committee of Weihai Central Hospital Affiliated to the Medical College of Qingdao University.

**Table 1:** The relationship between miR-33a-5p expression and clinicopathological characteristics among patients with PDAC

Characteristics	Cases (n = 58)	miR-33a-5p expression		*P*-values
		Low (n = 29, median ≤ 0.865)	High (n = 29, Median > 0.865)	(chi-square test)
Age				0.421
< 60 year	35	19	16	
≥ 60 year	23	10	13	
Gender				0.291
Male	32	18	14	
Female	26	11	15	
Tumor size				0.588
< 4 cm	36	17	19	
≥ 4 cm	22	12	10	
TNM stage				0.012*
I/II	39	15	24	
III/IV	19	14	5	
Lymph node metastasis				0.036*
Negative	43	18	25	
Positive	15	11	4	
Differentiation				0.599
Well/moderate	30	16	14	
Poor	28	13	15	

*p < 0.05

### Cell culture

We purchased four PDAC cell lines (BxPC-3, ASPC-1, SW1990 and PANC-1) and the normal pancreatic cell line HPNE from American Type Culture Collection. RPMI-1640 medium (Gibco, Grand Island, NY, USA) was used to culture all PDAC cell lines. HPNE cells were cultured in Dulbecco's modified Eagle medium (DMEM) containing 10% FBS (Gibco). All media were supplemented with 10% fetal bovine serum (FBS, Gibco) at 37 °C under a humidified atmosphere with 5% CO_2_.

### Cell transfection

MiR-33a-5p mimics, inhibitor and corresponding negative control (miR-NC and inhibitor NC, respectively) were obtained from GenePharma Co., Ltd. (Shanghai, China). The open reading frame of the human RAP2A gene was amplified and cloned into the pcDNA3.0 vector to construct the RAP2A overexpression plasmid (pcDNA3.0-RAP2A) by RiboBio Co., Ltd. (Guangzhou, China). Meanwhile, the empty pcDNA3.1 vector was used as the NC. For miR-33a-5p overexpression, SW1990 and PANC-1 cells at a density of 8 × 10^5^ cells per well were seeded into six-well plates, followed by transfection with miR-33a-5p mimics or miR-NC. For miR-33a-5p knockdown, miR-33a-5p inhibitor or inhibitor NC was transfected into BxPC-3 and ASPC-1 cells at a density of 8 × 10^5^ cells per well in six-well plates. For RAP2A overexpression, SW1990 and PANC-1 cells were pcDNA3.1-RAP2A or pcDNA3.1. In the rescue experiments, co-transfection with miR-33a-5p mimics and pcDNA3.1-RAP2A or pcDNA3.1 was performed in SW1990 and PANC-1 cells. Lipofectamine 2000 (Invitrogen, Grand Island, NY, USA) was utilized to perform all transfection procedures.

### Reverse-transcription quantitative PCR (RT-qPCR)

We extracted the RNA sample from tissue samples or cell lines with the TRIzol Reagent (Thermo Fisher Scientific, Waltham, MA, USA). For miR-33a-5p analysis, reverse transcription was carried out with the TaqMan MicroRNA Reverse Transcription Kit (Applied Biosystems, Foster City, CA, USA). Next, we determined the miR-33a-5p expression level using the TaqMan MicroRNA PCR Kit (Applied Biosystems) with U6 as the internal control. For analysis of RAP2A mRNA level, cDNA was chemically synthesized with the PrimeScript RT-PCR Kit (TaKaRa, Dalian, China). The RAP2A mRNA expression level was measured using the SYBR-Green Master Mix (Takara) with GAPDH as the loading control. The 2^−ΔΔCq^ method was used to performed quantitation of miR-33a-5p or RAP2A levels [25].

### CCK-8 assay

Approximately 4,000 cells were seeded into the each well of 96-well plates and cultured overnight. At 0, 24, 48 and 72 h after inoculation, cells in each well were incubated with 10 µL of CCK-8 (Cell Counting kit-8) solution (Dojindo, Kumamoto, Japan) for an additional 2 h at 37 °C. We then used a microplate reader to determine the optical density (OD) value in each well. All experiments were performed in triplicate.

### Migration and invasion assay

For the cell migration assay, we first suspended the transfected cells (1.0 × 10^4^) with serum-free medium and then plated them into the upper Transwell chamber (Corning Inc., Corning, NY, USA). Meanwhile, we put 500 µL of medium supplemented with 20% FBS as a chemoattractant into the lower chamber. After incubation for 24 h, the cells in the lower chamber were fixed with 100% methanol and stained with 0.1% crystal violet and counted under a light microscope in five randomly selected fields. The cell invasion assay was performed using a similar method as the migration assay, except that the chambers were precoated with Matrigel (BD Biosciences). All experiments were performed in triplicate.

### Luciferase reporter assay

To confirm the association between miR-33a-5p and RAP2A, the predicted miR-33a-5p-binding sequences at 3ʹ-UTR fragments of RAP2A as wild-type (WT) and the corresponding mutant 3ʹ-UTR fragments of RAP2A as MUT were chemically synthesized by GenePharma Co., Ltd. (Shanghai, China). These fragments were inserted into pmirGlo Dual-Luciferase Vectors (Promega; Carlsbad, CA, USA) to generate WT and MUT RAP2A constructs, respectively. Next, SW1990 and PANC-1 cells were co-transfected with 100 μg/mL pmirGlo WT or MUT RAP2A construct and 50 nmol/L miR-33a-5p mimics or miR-NC by means of Lipofectamine 2000. Forty-eight hours after transfection, we measured the firefly and Renilla luciferase activities with a Dual-Luciferase Reporter Assay System (Promega) and calculated the ratio of firefly activity/Renilla activity to obtained relative luciferase activity.

### RNA immunoprecipitation (RIP) assay

We used the Magna RIP RNA Binding Protein Immunoprecipitation Kit (Millipore, USA) to perform the RIP assay for verifying the association between RAP2A and miR-33a-5p. In brief, RIP lysis buffer was first used to lyse SW1990 and PANC-1 cells. Then, obtained cell lysate was treated with magnetic beads conjugated with anti-Argonaute2 (Ago2) antibody (Abcam, Cambridge, MA, USA) or negative control IgG in RIP buffer. Subsequently, each sample was incubated with proteinase K to digest proteins. Finally, RNAs were purified and validated by reverse-transcription quantitative PCR.

### Western blot analysis

The protein sample was prepared with Radio-Immunoprecipitation Assay (RIPA) buffer (Pierce Biotechnology, IL, USA) and quantified with the BCA method. Then, thirty micrograms of protein sample were separated by 10% SDS-PAGE gels, which were next transferred onto PVDF membranes. Then 5% non-fat milk diluted in TBS containing 0.05% Tween 20 (TBST) was used to block the membranes. Incubation of primary antibodies against RAP2A and GAPDH was performed overnight at 4 °C, followed by further incubation with horseradish peroxidase-conjugated secondary antibody. Finally, we used an enhanced chemiluminescence kit (Pierce Biotechnology) to visualize the target proteins.

### Meta-analysis based on Oncomine microarray database

To obtain the expression pattern of RAP2A in pancreatic cancer, we searched the Oncomine database (www.onocomine.org) by setting the key words: “RAP2A”, “Cancer vs. Normal Analysis”, “Pancreatic Cancer” and “mRNA” to conduct a meta-analysis of RAP2A expression in pancreatic tissue vs. normal tissue.

### Statistical analysis

After data analysis with GraphPad Prism software (La Jolla, CA, USA), we presented all data as mean ± standard deviation of three independent experiments. The possible correlation between miR-33a-5p expression (the median was used as a cutoff value) and clinical categorical parameters was investigated with the chi-square test. The association between miR-33a-5p and RAP2A mRNA levels was assessed by Spearman correlation analysis. Evaluation of differences between groups was performed with Student’s t-test or one-way analysis of variance. Statistical significance was accepted when a *p* value was less than 0.05.

## Results

### Expression of miR-33a-5p and its association with clinicopathological features in PDAC

**Fig. 1:**
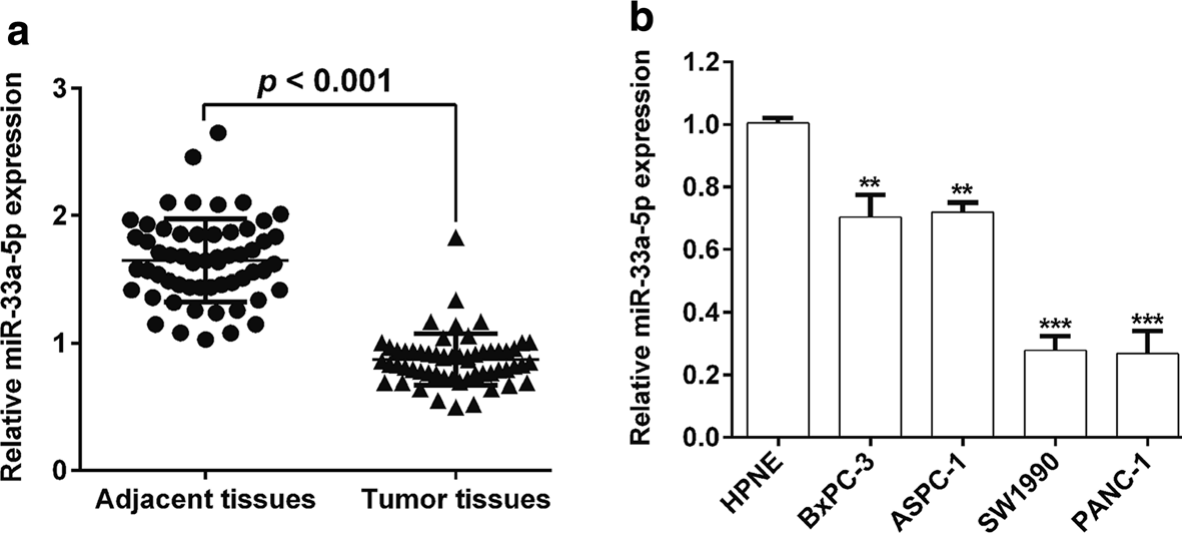
Downregulated miR-33a-5p in PDAC. **A** We performed RT-qPCR analysis to determine miR-33a-5p expression level in paired PDAC tissue samples and adjacent pancreatic tissues (n = 58). **B** We performed RT-qPCR analysis to measure miR-33a-5p expression level in BxPC-3, ASPC-1, SW1990, PANC-1 and HPNE cell lines. **p < 0.01, ***p < 0.001 vs. HPNE

We performed RT-qPCR to determine the miR-33a-5p expression in PDAC tissue samples. The results showed that miR-33a-5p expression level was significantly lower in PDAC tissues than in matched adjacent tissues (Fig. 1A). Likewise, miR-33a-5p expression was obviously lower in BxPC-3, ASPC-1, SW1990 and PANC-1 than in the normal pancreatic cell line HPNE (Fig. 1B). Then, we divided all patients into a low miR-33a-5p expression group (n = 29) and a high miR-33a-5p expression group (n = 29) by the median miR-33a-5p expression. As shown in Table 1, reduced miR-33a-5p was significantly associated with TNM stage (*p* = 0.012) and lymph node metastasis (*p* = 0.036).

### MiR-33a-5p inhibited PDAC cell proliferation, migration and invasion in vitro

**Fig. 2:**
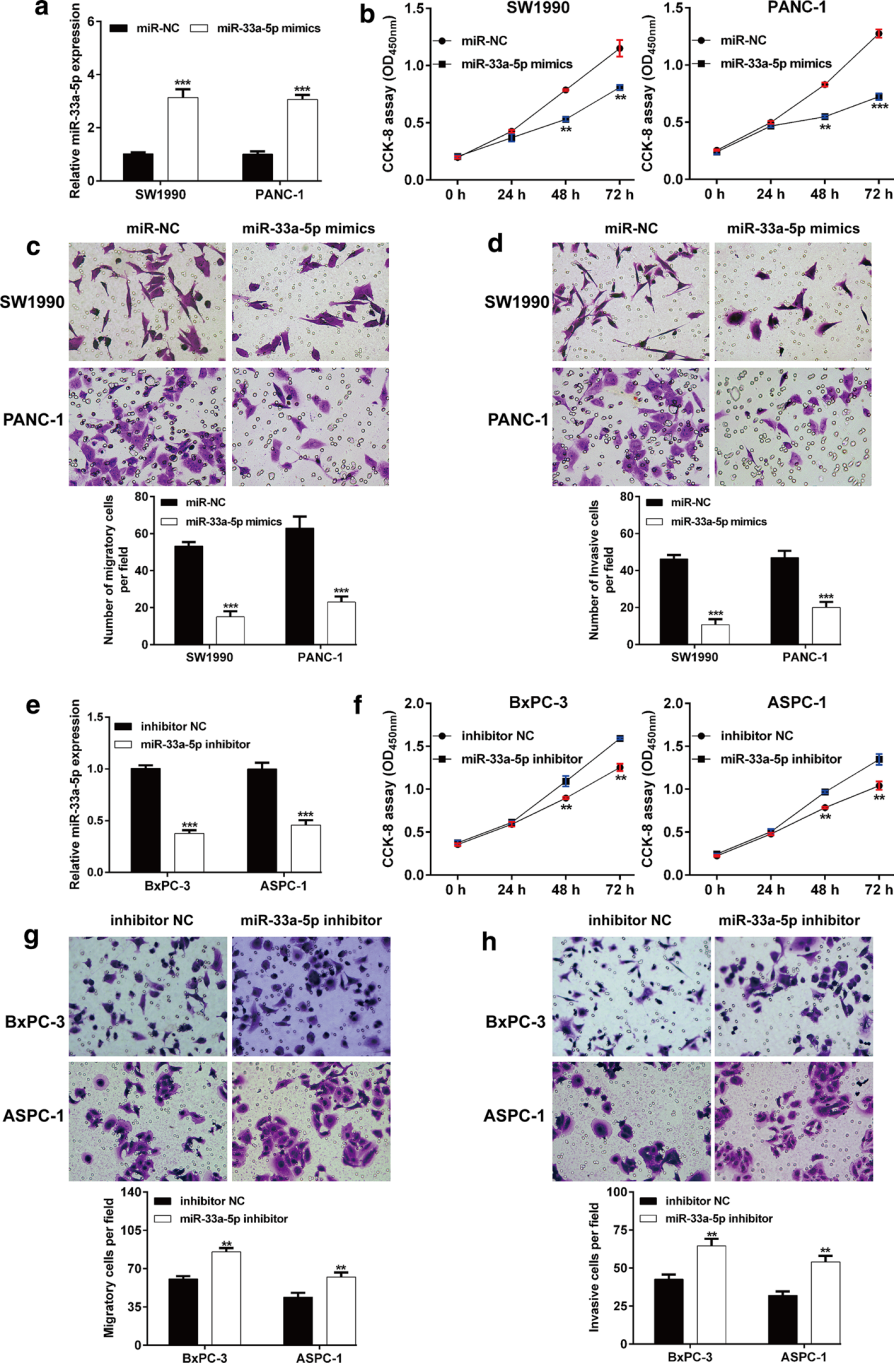
MiR-33a-5p exerted suppressive effects on PDAC cell proliferation, migration and invasion. **A** Expression level of miR-33a-5p was assessed by RT-qPCR in SW1990 and PANC-1 cells after transection with the miR-33a-5p mimics or miR-NC. **B** CCK-8 assay was used to evaluate changes in SW1990 and PANC-1 cell proliferation following transfection with miR-33a-5p mimics or miR-NC. **C**, **D** Cell migration and invasion were analyzed in SW1990 and PANC-1 cells following transfection with miR-33a-5p mimics or miR-NC. **E** BxPC-3 and ASPC-1 cells were transected with the miR-33a-5p inhibitor or miR-NC. After that, the expression level of miR-33a-5p was assessed by RT-qPCR. **F** Changes in proliferation of transfected BxPC-3 and ASPC-1 cells were evaluated by the CCK-8 assay. **G**, **H** Cell migration and invasion were analyzed in transfected BxPC-3 and ASPC-1. **p < 0.01, ***p < 0.001 vs. miR-NC or inhibitor NC group

Considering the relatively low miR-33a-5p expression in SW1990 and PANC-1 cell lines, we selected SW1990 and PANC-1 cells lines to perform gain-of-function assays to investigate the potential biological function of miR-33a-5p in vitro. As shown in Fig. 2A, miR-33a-5p mimic transfection obviously overexpressed miR-33a-5p expression in SW1990 and PANC-1 cells, compared with miR-NC transfection. Then, the role of miR-33a-5p in cell proliferation and metastasis in vitro was analyzed using CCK-8 assay and transwell assay. It was observed that miR-33a-5p mimic transfection significantly impaired the cell proliferation ability in both SW1990 and PANC-1 cells as compared with transfection with miR-NC (Fig. 2B). In addition, overexpression of miR-33a-5p significantly decreased the migratory (Fig. 2C) and invasive (Fig. 2D) abilities. To further confirm the suppressive effects of miR-33a-5p on PDAC cells, we performed loss-of-function assays in BxPC-3 and ASPC-1 cell lines. RT-qPCR was first used to validate the knockdown of miR-33a-5p after transfection with miR-33a-5p inhibitor (Fig. 2E). Contrary to miR-33a-5p overexpression, we found that knockdown of miR-33a-5p significantly promoted cell proliferation (Fig. 2F), migration (Fig. 2G) and invasion (Fig. 2H) abilities in BxPC-3 and ASPC-1 cells. Collectively, these data indicated the tumor suppressive role of miR-33a-5p in PDAC cell growth and metastasis in vitro.

### miR-33a-5p downregulated RAP2A expression by directly targeting its 3′-UTR

**Fig. 3:**
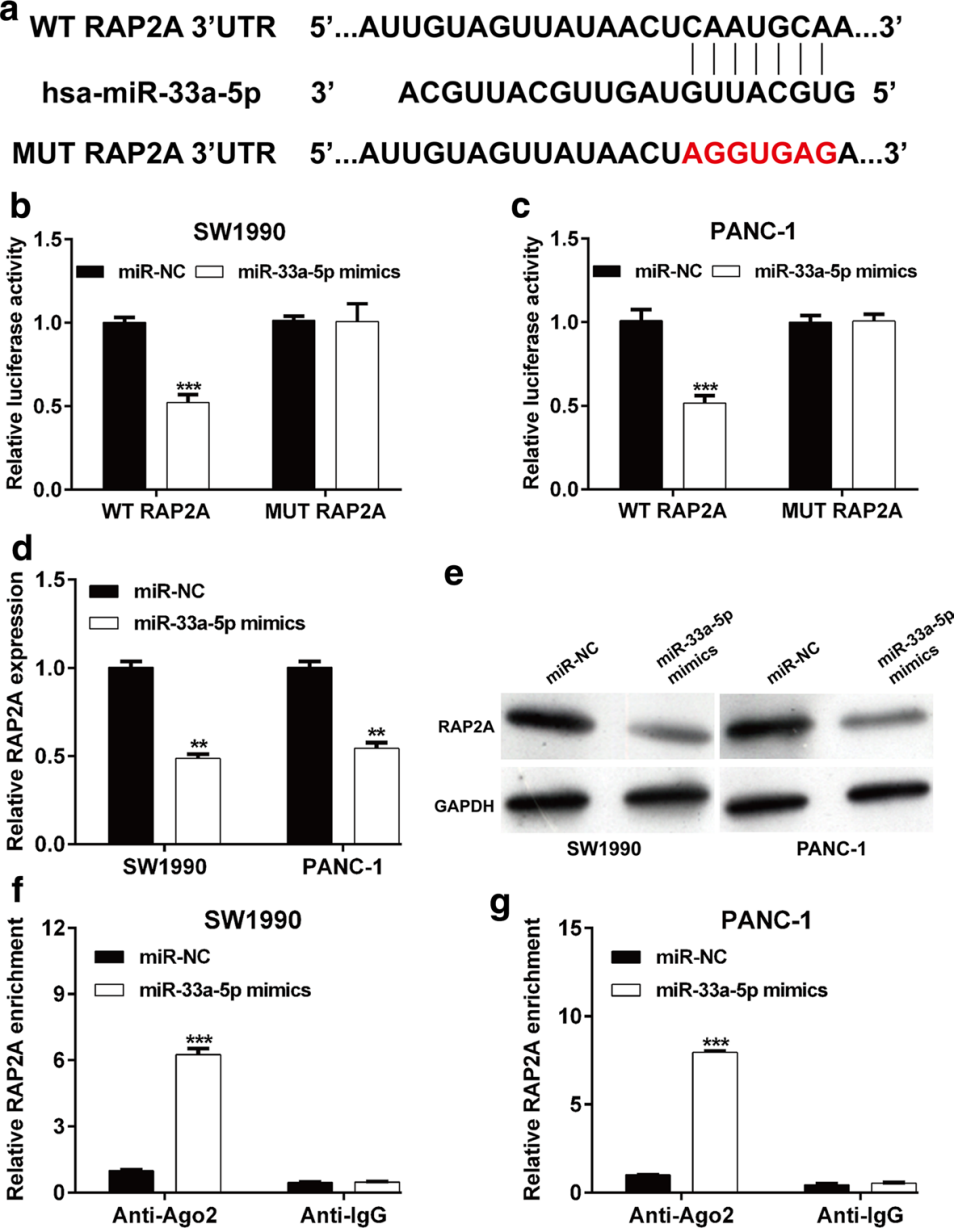
RAP2A was the target of miR-33a-5p. **A** Binding sequences of miR-33a-5p at the RAP2A 3′-UTR mRNA are displayed. **B**, **C** Relative luciferase activity was determined after co-transfection with either miR-33a-5p mimics or miR-NC and either WT RAP2A or MUT RAP2A in SW1990 and PANC-1 cells. **D**, **E** RT-qPCR and western blotting were performed to determine RAP2A expression level in SW1990 and PANC-1 cells following transfection with miR-33a-5p mimics or miR-NC. **F**, **G** RNA RIP experiments were performed in SW1990 and PANC-1 cells and the co-precipitated RNA was analyzed by RT-qPCR. **p < 0.01, ***p < 0.001 vs. miR-NC group

Next, we determined the association between miR-33a-5p and RAP2A. Using TargetScan (http://www.targetscan.org/), we found a highly conserved miR-33a-5p-binding site at the 3′-UTR of RAP2A (Fig. 3A). Moreover, the results from the luciferase reporter assay revealed that miR-33a-5p overexpression significantly suppressed the luciferase activity of the plasmid carrying WT RAP2A luciferase construct but not the MUT RAP2A construct in SW1990 (Fig. 3B) and PANC-1 (Fig. 3C) cells. Furthermore, forced miR-33a-5p expression decreased RAP2A expression at both mRNA (Fig. 3D) and protein (Fig. 3E) levels. To explore the associations between RAP2A and miR-33a-5p, RIP assay was performed on SW1990 and PANC-1 cell extracts with incubation with the Ago2 antibody. The results showed that RAP2A was significantly enriched in the miR-33a-5p mimic-transfected cells (Fig. 3F, G). These data suggested that miR-33a-5p directly targeted RAP2A in PDAC cells.

### miR-33a-5p inversely correlated with RAP2A levels in PDAC tissues

**Fig. 4:**
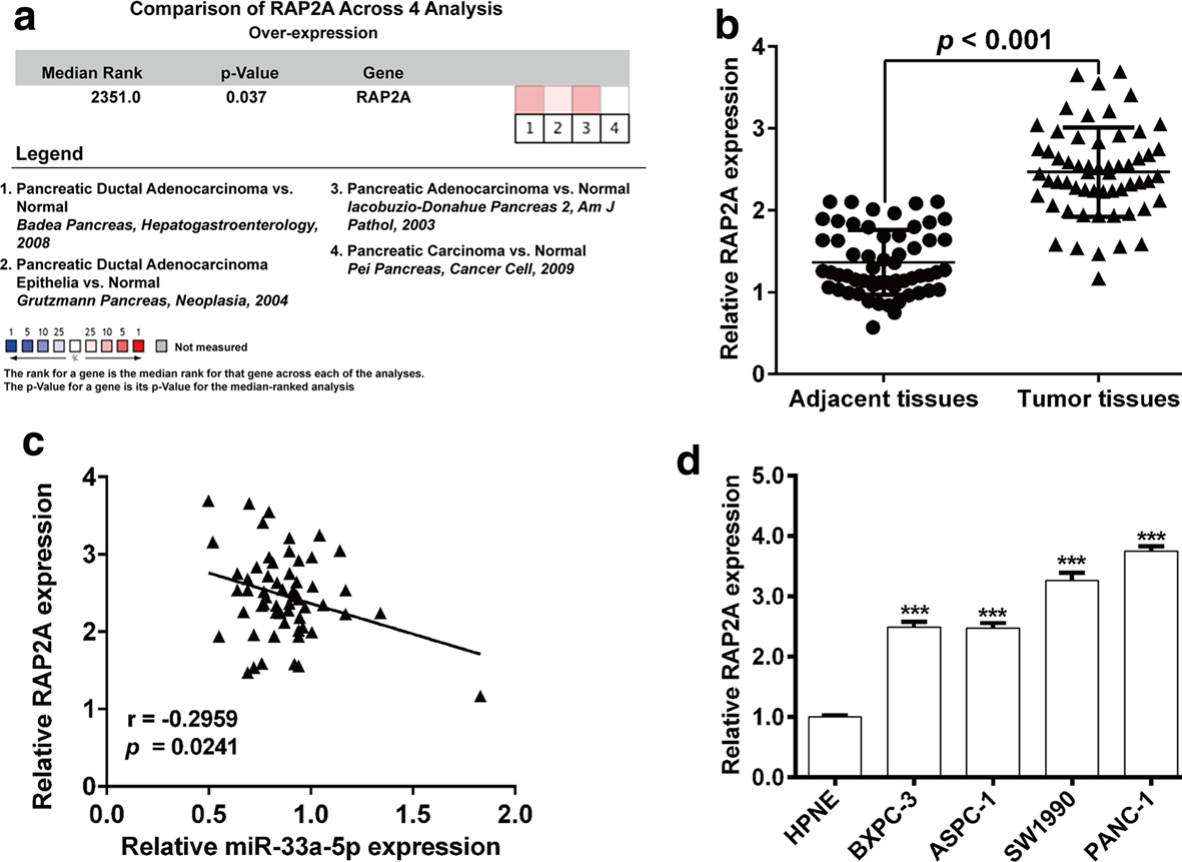
RAP2A was inversely correlated with miR-33a-5p expression in PDAC. **A** Our meta-analysis included four microarray datasets regarding RAP2A mRNA expression in pancreatic cancer vs. normal tissues. Data are shown as the median rank of RAP2A through each dataset analysis. *P*-value for RAP2A was presented using the median ranked analysis for pancreatic cancer vs. normal tissues. **B** RAP2A mRNA levels analyzed using RT-qPCR in paired PDAC tissue samples and adjacent pancreatic tissues (n = 58). **C** An inverse association was identified between miR-33a-5p and RAP2A mRNA expression levels through Spearman correlation analysis (r = − 0.2959, *p* = 0.0241). (D) RAP2A mRNA levels were determined in BxPC-3, ASPC-1, SW1990, PANC-1 and HPNE cell lines. ***p < 0.001 vs. HPNE

Subsequently, we searched the online Oncomine database to investigate the expression pattern of RAP2A in pancreatic cancer. As shown in Fig. 4A a total of four online microarray datasets—Badea Pancreas, Grutzmann Pancreas, lacobuzio-Donahue Pancreas 2 and Pei Pancreas—were included in our study, which consistently indicated that RAP2A mRNA level was significantly overexpressed in pancreatic cancer tissues (gene median rank: 2351.0, *p* = 0.037). Subsequently, we found that RAP2A mRNA expression was noticeably elevated in PDAC tissue samples, in comparison with normal pancreatic tissue samples (Fig. 4B). Moreover, we observed an inverse correlation between miR-33a-5p and RAP2A among PDAC tissue samples (Fig. 4C,  r= − 0.2959, *p* = 0.0241). In addition, we analyzed the expression of RAP2A mRNA in PDAC cell lines. As expected, the expression of RAP2A was obviously higher in PDAC cell lines (BxPC-3, ASPC-1, SW1990 and PANC-1) than that in the normal pancreatic cell line HPNE (Fig. 4D).

### Overexpression of RAP2A promoted PDAC cell proliferation, migration and invasion

**Fig. 5:**
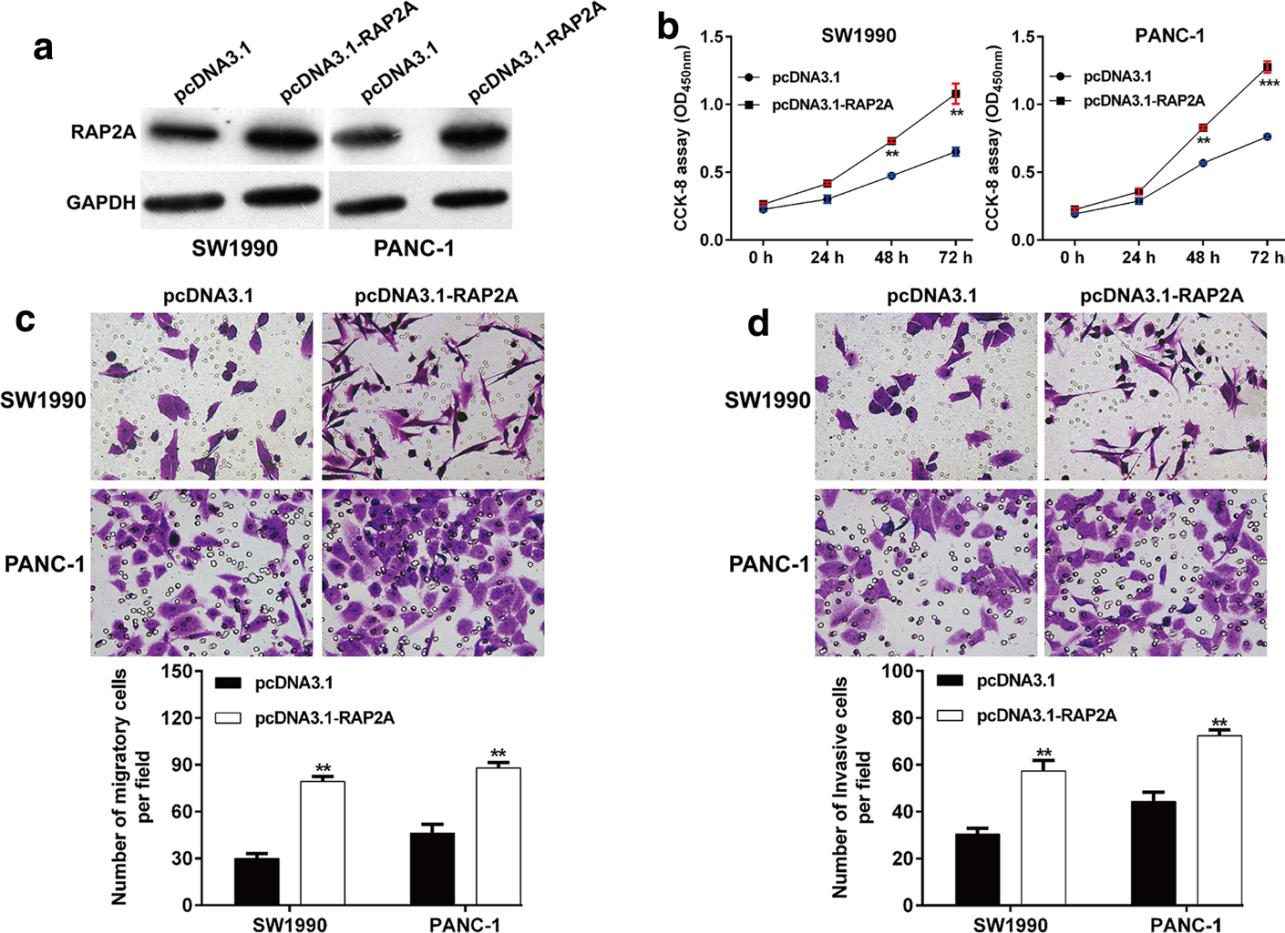
Regulatory effects of RAP2A overexpression on PDAC cell proliferation, migration and invasions. **A** Transfection of pcDNA3.1-RAP2A or pcDNA3.1 was performed in SW1990 and PANC-1 cells. After that, the protein expression level of RAP2A was measured by western blot analysis. **B** Changes in proliferation of transfected SW1990 and PANC-1 cells were evaluated by CCK-8 assay. **C**, **D** Cell migration and invasion were analyzed in transfected SW1990 and PANC-1 cells. **p < 0.01, ***p < 0.001 vs. pcDNA3.1 group

Next, transfection with pcDNA3.1 or pcDNA3.1-RAP2A was carried out in SW1990 and PANC-1 cells. As expected, transfected pcDNA3.1-RAP2A obviously upregulated the protein level of RAP2A in PDAC cells (Fig. 5A). In contrast to miR-33a-5p overexpression, RAP2A overexpression significantly enhanced cell proliferation ability in SW1990 and PANC-1 cells (Fig. 5B). The results from the transwell assay indicated that overexpression of RAP2A remarkedly increased the number of migratory (Fig. 5C) and invasive (Fig. 5D) cells in SW1990 and PANC-1 cells.

### Overexpression of RAP2A attenuated the tumor suppressive effects of miR-33a-5p on PDAC cells

**Fig. 6:**
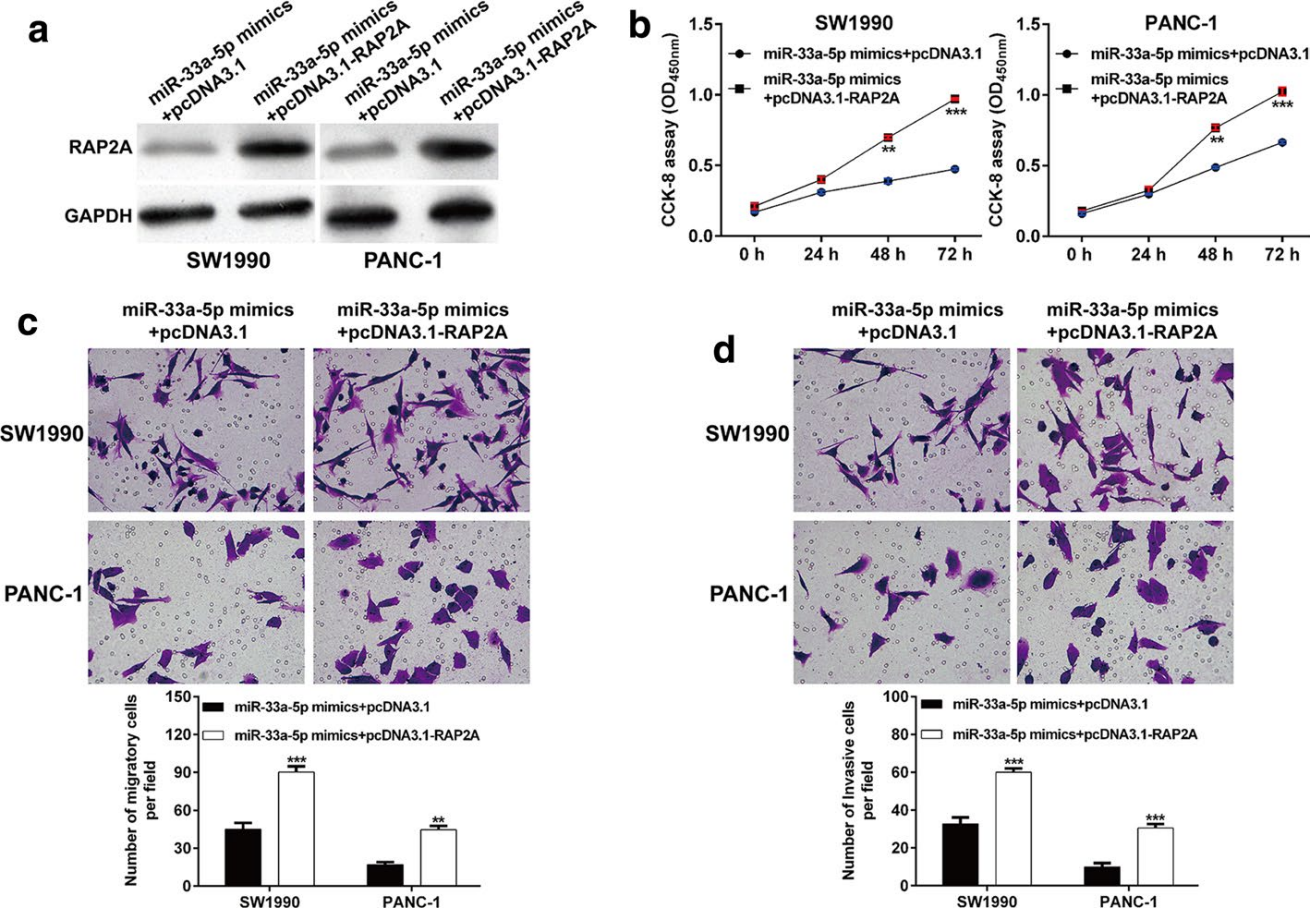
RAP2A reintroduction attenuated the effects of miR-33a-5p overexpression. SW1990 and PANC-1 cells were co-transfected with the miR-33a-5p mimics and either pcDNA3.1-RAP2A or pcDNA3.1. **A** RAP2A protein expression was determined using western blot analysis in the aforementioned cells. The transfected cells were subjected to the CCK-8 assay (**B**), Transwell migration (**C**) and invasion (**D**) assays, respectively. **p < 0.01, ***p < 0.001 vs. the miR-NC + pcDNA3.1 group

To confirm whether the suppressive effects of miR-33a-5p in PDAC cells were mediated by the repression of RAP2A, we carried out rescue experiments. Co-transfection with the miR-33a-5p mimics and either pcDNA3.1-RAP2A or the empty vector pcDNA3.1 was performed in SW1990 and PANC-1 cells. As depicted in Fig. 6A, pcDNA3.1-RAP2A mimic transfection reversed the decreased RAP2A protein level in SW1990 and PANC-1 cells after miR-33a-5p mimic transfection. In functional analysis, CCK-8 assay showed that overexpression of RAP2A expression partially reversed the suppressive effects of miR-33a-5p on cell proliferation in SW1990 and PANC-1 (Fig. 6B). Similarly, restoration of RAP2A reversed the suppressive effects of miR-33a-5p overexpression on cell migration (Fig. 6C) and invasion (Fig. 6D) in SW1990 and PANC-1 cells. These findings indicated that miR-33a-5p exerted suppressive effects on PDAC cells at least partially targeting RAP2A.

## Discussion

Here, we found that miR-33a-5p expression level was significantly downregulated in PDAC tissues and cell lines. Low miR-33a-5p level expression was associated with TNM stage and lymph node metastasis in PDAC patients. Consistently, miR-33a-5p was downregulated in colorectal cancer [16], melanoma [26] and osteosarcoma [14]. Decreased miR-33a-5p expression was closely associated with poor prognosis in ESCC [15]. Downregulation of miR-33a-5p was positively correlated with malignancy and poor prognosis in glioma patients [27]. These facts further indicated that miR-33a-5p might exert suppressive effects on PDAC cells.

Our further functional assay revealed that miR-33a-5p significantly suppressed the proliferation, migration and invasion of PDAC cells. Similarly, upregulation of miR-33a-5p attenuated osteosarcoma cell growth in vitro [14] and suppressed hepatocellular carcinoma tumorigenesis [28]. Notably, miR-33a-5p significantly inhibited cell growth and insulin production of pancreatic β-cells in gestational diabetes mellitus, which might imply a correlation between miR-33a-5p and the pathogenesis of PDAC [29].

Furthermore, RAP2A, a member of the GTP-binding protein family, was validated as a direct target of miR-33a-5p in PDAC cells. RAP2A has been reported to be upregulated in various types of human cancer, such as lung cancer [21] and nasopharyngeal carcinoma [22]. In line with this, our data showed that RAP2A expression was overexpressed in PDAC tissues and inversely correlated with miR-33a-5p expression. Moreover, we observed that overexpression of RAP2A attenuated the tumor suppressive effects of miR-33a-5p on PDAC cell proliferation, migration and invasion in both SW1990 and PANC-1 cells, which suggested that miR-33a-5p suppressed the malignancy of PDAC cells in vitro possibly through directly targeting RAP2A. Combined with the oncogenic role of RAP2A in tumor cells [24], including renal cell carcinoma [23] and thyroid cancer [30], we thus speculated that RAP2A was the downstream regulator involved in the miR-33a-5p negative regulation of PDAC cell functions. Considering that the association between miR-33a-5p and RAP2A has not been reported before, we believe that identification of the miR-33a-5p/RAP2A axis may be important for understanding the progression of PDAC.

## Conclusions

In summary, our findings illustrated that miR-33a-5p acts a potential tumor suppressor in PDAC progression by targeting RAP2A, which provides a potential therapeutic strategy against PDAC. However, further investigations, including the effects of miR-33a-5p on the metastasis of PDAC cells in vivo, are still needed to fully elucidate the mechanism of their interaction in PDAC.

## Data Availability

The data are available from the corresponding author upon request.

## Abbreviations

PDAC: Pancreatic ductal adenocarcinoma
RT-qPCR: Reverse-transcription quantitative PCR
3′-UTRs: 3′-Untranslated regions
RIP: RNA immunoprecipitation
ATCC: American Type Culture Collection
CK-8: Cell Counting Kit -8
OD: Optical density
WT: Wild-type
HRP: Horseradish peroxidase
